# Salmagundi

**Published:** 1885-03

**Authors:** 


### SALMAGUNDI.

EDITED BY E. G. BETTY, D. D. S.

THE man does not live who *is* just what he would *like* to be.

A FEE in the hand is worth two in the book, says the New York *Medical Record*.

TWELVE manufacturers in the United States turn out 10,000,000 store-teeth annually.

The output of gold from the mines of the United States in 1884, amounted to nearly $31,000,000.

---

Boy; gun.
Gun bust,
Boy done.

---

The loneliest doctor in the world is the opthalmologist who has made no report of experience in the use of cocaine.—*Medical Record.*

---

It is said that Admiral Courbet, commander of the French blockading squdron at Formosa, has "a pleasant smile, deprived of some of its warmth by the display of unskillful dentistry."

---

As a general statement, the finer the nervous organization, the more precocious, or brilliant, the intellect, the greater will be the tendency to dental deformity. Kingsley.

---

There is an old dentist in Washington who boasts that he has pulled over 100,000 teeth by actual count, and by the aid of a pair of pinchers, of course. He expects as a monument an immense marble image of a tooth, big enough to cover an acre.—*Daily Paper.*

---

Dr. Morgan Hughes (*British Medical Journal*) has succeeded in relieving severe toothache by the application of a ten per cent. solution of hydrochlorate of cocaine in oil of cloves, applied freely by means of pledgets of cotton wool, both to the pulp cavity and to the gums.

---

The thigh bones of cattle, minus the knuckles, are worth $80 per ton, and are manufactured into handles for tooth brushes. Very few of these bones are exported, which shows that America does not depend upon London and Paris for tooth brushes so much as formerly.

Dr. Miller, of Berlin, reports "a case in practice" in the January number of the *Independent Practitioner*, for January, in which he states that the patient, Frau X, often complained of "having a taste of old Limburg cheese in her mouth." Nothing wonderful in that; it's to be expected from that quarter of the globe.

---

Neuralgia and headache may be successfully relieved by ten or fifteen grain doses of salicylate of sodium every hour. Generally one dose is sufficient; severe cases sometimes requiring two or three doses. Dr. Quessenberry, of Worthington, Ky., is authority for the above, and says it is superior to quinine, bromide of potassium, morphia, etc.

---

An ingenious Frenchman has perfected an invention for reshaping noses, the device to be worn at night, and so adjusted that the nose may be pressed into any desirable shape. This invention might be used by the go-ahead dentist to enhance the effects of his prosthetic efforts to produce a beautiful face out of indifferent material and thereby increase his exchequer.

---

"The famous sheeps-head fish of the Atlantic seaboard is one of the finest articles of diet offered by salt water, and is eagerly sought after by epicures. There have recently been some magnificent ones on sale in Fulton Market, New York. The fish obtains its name from having a set of upper and lower teeth that are really human in their shape and appearance."

---

The publishers of the *British Journal of Dental Science* announce on their card that their journal is the "*oldest established dental journal in the world.*" Shades of Ananias! Where does the Register come in? The *B. J. D. S.* was established *twenty-seven* years ago, while the Register issued its first number in 1847, and has since continued to carry on the good work. The publishers of the *B. J. D. S.* would better study the chronology of the profession a little before they make any more such rash statements.

We published a paragraph from a daily paper in last month's number, relating to a case of Dr. Kirk's, in which he had administered nitrous-oxide. This was done with the hope that it would induce a true statement of existing lesions, as a hasty telegram does not always embody the facts as they exist. The *Ohio State Journal*, for February, contains Dr. Kirk's account, as requested by Dr. Watt. The hemorrhage was simply from the alveolus, and the other symptoms those of hysteria. The case is interesting, and Dr. Kirk's letter is worthy of perusal.

---

"The statistics as to the use of opium are almost startling. In 1840 about 20,000 pounds of opium were consumed in the United States; in 1880, 533,450 pounds. In 1868 there were about 90,000 habitual opium eaters in the country; now they number over 500,000. If the number of opium eaters is to be doubled every six or seven years, the opium habit will soon become almost as universal as the whisky habit, and far more baneful in its effects. More women than men are addicted to the use of opium. Dr. Hammond places the proportion at three to one."

---

"The Japanese dentist performs all his operations of tooth drawing with the thumb and fore finger of one hand, and thus he never terrifies his patients with an array of steel instruments. The skill necessary to do this is only acquired by long practice, but once it is obtained the operator is able to extract about half a dozen teeth in thirty seconds without once removing his fingers from the patient's mouth. The dentist's education commences with the pulling out of pegs that have been pressed into soft wood; it ends with the drawing of hard pegs which have been driven into an oak plank with a mallet. It is said that no human jaw can resist the delicate but powerful manipulation of the Japanese dentist."

---

Mary was a buxom country lass, and her father was an upright deacon in a Connecticut village. Mary's plan of joining the boys and girls in a nutting party was frustrated by the unexpected arrival of a number of the "brethren" on their way to conference, and Mary had to stay at home and get dinner for her father's clerical guests. Her already ruffled temper was increased by the reverend visitors themselves, who sat about the stove and in the way. One of the good ministers noticed the wrathful impatience, and, desiring to rebuke the sinful manifestations, said sternly: "Mary, what do you think will be your occupation in hell?" "Pretty much the same as it is on earth," she replied, "cooking for ministers."

---

PROFESSOR TYNDALL thus endeavors to explain the immunity obtained against a second attack of a contagious disease.

"One of the most extraordinary and unaccountable experiences in medicine was the immunity secured by a single attack of a communicable disease against future attacks of the same malady. Small-pox, typhoid, or scarlatina, for example, was found, as a general rule, to occur only once in a life-time of the individual, the successful passage through the disorder apparently rendering the body invulnerable. Reasoning from analogy, I have ventured to express the opinion that the rarity of second attacks of communicable disease was due to the removal from the system, by the first parasitic crop, of some ingredient necessary to the growth and propagation of the parasite."

---

AMONG the more recent attempts to establish a communication between the stomach and intestines is the novel case of Dr. Conner, of Cincinnati, which is recorded in the *Medical News* for Nov. 22, 1884. The entire stomach was extirpated "with the hope of attaching the cardiac end to some portion of the intestinal tract, so that the fluids poured out in the upper part of the small intestine might flow down to meet the food and cause digestion in the part of the intestine where they come together." As the patient died upon the table the operation was not completed. However feasible it may be, so far as surgical procedures are concerned, we fail to see that, in the event of recovery, it could be of any service, from the fact of the supply of the gastric juice being entirely cut off. Hence we trust that the operation will not find imitators.

